# Supplementary material for: Impact of changes in chatbot’s facial expressions on user attention and reaction time
Source: PLoS One. 2023 Jul 27;18(7):e0288122. doi: 10.1371/journal.pone.0288122 (PMC10374122; doi:10.1371/journal.pone.0288122)
Supplement: Attachment — Submitted filename: PLOS___faces_review_2.pdf [file pone.0288122.s001.pdf]

# Response to Reviewer 2 Comments

March 2, 2023

At the beginning, we would like to thank the Reviewer for valuable comments and tips. We appreciate the time spent and helpful remarks. Please find the answers and comments to the doubts presented in the review enclosed below.

Point 1: the sample should be described with more details (es. gender, inclusion and exclusion criteria)

Response 1: Regarding gender, 50% of study participants were female, and 50% were male. Inclusion criteria could be defined as ”participants with normal or corrected-to-normal vision and between 18 to 35 years old”. Exclusion criteria could be defined as ”participants with a history of eye disease or neurological disorder, or any other condition that could affect the results of the study”.

Point 2: some captions and figures are unclear and are missing measurement units (e.g. ms)

Response 2: We have corrected that throughout the article.

Point 2a:fig 2 B the color code of the chart and the legend of the two histograms on top and on the right should be included

Response 2a: We have corrected this.

Point 2b:fig 2 C does not look like a “scatterplot”, I suggest an alternative definition. Furthermore, a better definition of “reaction” is needed and the caption is unclear.

Response 2b:We have corrected this. The reaction is pressing the mouse button when there was a change of the object (in our task, the photo with the expressed emotion) in the chatbot that appeared in the peripheral area.

Point 2c: fig 3 A in the caption “shows two groups of emotions (positive and negative) along with the coverage of individual groups of emotions” should be

more explicit; a description of the light green and light red should be provided also in a legend

Response 2c: We have corrected this. Positive emotions are a group of positively associated emotions. Negative emotions, in this case, are ”non-positive” emotions. Hence, they are in opposition to one another.

Point 2d: fig 3 B the color code of the chart and the legend of the two histograms on top and on the right should be included

Response 2d: We have corrected this.

Point 3a: “In fact, Mori observed an increased sensitivity to defects in a human-like form”: which type of defects are they referring to?

Response 3a: Mori observed that the more humanlike an object is, the more more familiar it seems, but only until a certain point at which defects understood as subtle deviations from human norms cause them to look disturbing.

Point 3b: “Additionally, there is a positive relationship between human likeness and knowledge”: the meaning is obscure

Response 3b: The effect hypothesis is that humanoid objects that look almost, but not quite, like real people make observers experience eerie or strangely familiar sensations of revulsion or eerieness. In fact, Mori observed a degree of vulnerability to defects in near-human forms - an ”uncanny valley” in what otherwise follows the relationship between prediction and familiarity. Since then, research has focused on affect, but still little is known about how humanoid objects are actually perceived.

Point 3c: “In detection, however, while happiness and surprise are still well detected, fear is also a well detected expression”: there seems to be an error here

Response 3c: We have corrected the text.

Point 3d: “Both of these emotions can be classified as positive, while the others, which were also present in the study, i.e., anger, disgust, sadness, and fear, are negative or pejorative”: the phrase does not link with the one before

Response 3d: We have corrected the text.

Point 3e: An important aspect is to increase the naturalness of the chatbot’s communication with the user: the phrase does not link with the one before

Response 3e: We have corrected the text.

Point 3f: "McCrickard et al. discussed utility as a value provided by the peripheral system as a whole and did not directly manipulate utility as part of their experiment, whereas we consider utility as the meaning of the content of the individual gaps": the concept of utility should be better defined

Response 3f: We have developed that in the paper. In studying peripheral or secondary display information representations, many researchers focus only on information gained, without measuring the changes in primary task performance caused by these displays (Gray and Saltzman, 1998). Similarly, some studies create an unrealistic testing environment given that the distinction between reaction and comprehension is unclear. While reaction and comprehension tasks are often closely related, the two objectives may imply differences to notification system information design.

Point 4a: The graphic avatar in the chatbot field will have a different impact on a conversation than a conversation with a human and increases the fluency of conversation"

Response 4a: We have corrected the text.

Point 4b: "This secondary task is usually a given, with the task supported by the peripheral system being evaluated"

Response 4b:We have corrected the text.

Point 4c: "In real systems, the chatbot, as well as system messages or in-video-game messages, aimed at helping users or players, is targeted at a more accurate peripheral search and identification tasks while performing a central search"

Response 4c:We have corrected the text.

Point 4d: "Starting the analysis at the base, we can conclude in Figure 2 (A)"

Response 4d:We have corrected the text.

Point 4e: "It can be seen here that the vast majority of reaction times ranged from 0.36 to 0.52 s. In this interval, most reactions were to the emotion of surprise"

Response 4e:We have corrected the text.
