# Supplementary material for: Impact of changes in chatbot’s facial expressions on user attention and reaction time
Source: PLoS One. 2023 Jul 27;18(7):e0288122. doi: 10.1371/journal.pone.0288122 (PMC10374122; doi:10.1371/journal.pone.0288122)
Supplement: Attachment — Submitted filename: Response to Reviewers.pdf [file pone.0288122.s002.pdf]

Response to Reviewer 1 Comments

June 9, 2023

At the beginning, we would like to thank the Reviewer for valuable comments and tips. We appreciate the time spent and helpful remarks. Please find the answers and comments to the doubts presented in the review enclosed below.

Point 1: At p. 2/13 “The ’social presence’ theory [11] suggests that the degree of social presence in a communication medium can affect the perceived level of social presence in the interaction”. This sentence is unclear as the authors are saying that “the degree of social presence [. . .] can affect the level of social presence”, which is a tautology. The social presence theory suggests that the degree to which a medium (such as a chatbot) is designed to look and act more like a human, with features such as a human-like appearance, facial expressions, and gestures, positively impact the level of social presence perceived in the interaction. As a result, users may feel like they are engaging in a more natural and authentic conversation, as if they were talking to a real human instead of a machine.

Response 1: We agree with the Reviewer’s remarks and we have implemented their suggestions. We removed redundant sentence about tautology to. ” the degree of social presence [. . .] can affect the level of social presence”. We made a correction in the text regarding this fragment in the comment. The new fragment of the text we have included in the work is: ”The extent to which a medium, like a chatbot, is designed to resemble and behave like a human, incorporating elements such as a human-like appearance, facial expressions, and gestures, can significantly influence the perceived level of social presence during the interaction. Consequently, users may experience a heightened sense of engaging in a genuine and natural conversation, almost as if they were conversing with an actual human being rather than a machine.”

Point 2: At p. 2/13 “In fact, Mori [12] observed a degree of defect proneness in near-human forms an ”uncanny valley” in what otherwise follows from the relationship between anticipation and familiarity”. That is not entirely precise. The Uncanny Valley (UV) hypothesis refers to the relationship between an entity’s human-likeness and familiarity (not between anticipation and familiarity). The human-likeness/familiarity relationship is correctly discussed by the authors in

the following sentence “Mori observed that the more human-like a technological object such as a robot is, the more familiar it seems; however, this is true only up to a certain point, at which even subtle deviations from human norms can make the object look disturbing”. “Anticipation”, “perceptual difficulty”, as well as “increased sensitivity to defects in a human-like form”, mentioned in this paragraph, actually refer to different proposed explanations of the UV effect. First, “anticipation” plays a key role in the expectation violation explanation of the uncanny valley. The expectation violation explanation suggests that the negative response to an entity in the UV occurs when it violates the expectations that humans have for how a human-like entity should behave and interact based on their past experiences and knowledge of human behaviour. Second, the sentence “Perceptual difficulty in distinguishing between a human-like object and its human counterpart would produce negative affect” refers to the “categorization ambiguity” explanations of the UV. When an entity is difficult to categorize as either animate or inanimate because it possesses some but not all of the features of a living entity, this creates a sense of ambiguity, which can lead to discomfort and unease. Third, “Mori observed an increased sensitivity to defects in a human-like form”. The higher sensitivity to deviations from typical human norms for more human-like characters is part of the perceptual mismatch explanations, according to which the UV effect is caused by any perceptual mismatch in the digital entity’s appearance. In this case, as humans interact with entities that resemble humans to a greater degree, they become more attuned to the nuances of human behaviour and appearance. This heightened sensitivity can increase awareness of deviations (mismatches) from typical human norms. [If you wish to know more, please see Katsyri et al. (2015) “A review of empirical evidence on different uncanny valley hypotheses: support for perceptual mismatch as one road to the valley of eeriness”.] Here I would suggest the authors keep it simple by just referring to the relationship between human-likeness and familiarity, without digging into the different explanations. Furthermore, I would change the sentence “The hypothesis suggests that humanoid objects, including chatbots, which appear almost, but not exactly, like real human beings, may evoke strange or strangely familiar negative as well as positive feelings in observers” removing the reference to positive feeling, which creates confusion while talking about the UV effect. Finally, as observed by the other reviewer in the first step of revisions, the meaning of “Additionally, there is a positive relationship between human likeness and knowledge”: is unclear. I would suggest the author to remove it.

Response 2: We agree with the Reviewer’s remarks and we have implemented their suggestions. We removed redundant sentence. We’ve modified the Uncanny Valley Theory section, which we’ve addressed in the section below: ”The extent to which a medium, like a chatbot, is designed to resemble and behave like a human, incorporating elements such as a human-like appearance, facial expressions, and gestures, can significantly influence the perceived level of social presence during the interaction. Consequently, users may experience a heightened sense of engaging in a genuine and natural conversation, almost as if they

were conversing with an actual human being rather than a machine. The visualization of chatbots and their social features should target users' expectations to ultimately avoid frustration and dissatisfaction. The effects of an electronic conversation on human behavior and the perceived level of anthropomorphism are a part of the broad issue of human attitudes towards humanoid technologies. According to the theory proposed by Mori, the more a character resembles a human, the more it is accepted by us and evokes positive feelings. However, when a character becomes too realistic but still has subtle differences, such as unnatural movements or improper proportions, we experience a sense of unease or rejection in our minds. This is the moment when we enter the so-called Uncanny Valley. Mori's theory suggests that the acceptance of artificial figures increases with their level of realism until a certain point, after which there is a sudden decline in acceptance. Only when a character reaches an exceptionally high level of realism, almost indistinguishable from a living human, does acceptance increase again. The phenomenon of the Uncanny Valley has also been applied in the context of avatars with facial expressions. Irregularities in facial expression movements or inconsistencies with our expectations can make us feel uneasy and result in negative attitudes towards such avatars. Therefore, it is not surprising that the hypothesis regarding the Uncanny Valley has been adopted to explain the poor commercial success of some animated films in the media. In a study conducted by Katsyri et al. (2015), existing research on people's reactions to artificial figures with varying degrees of realism was analyzed to investigate which hypotheses best explain this phenomenon. One hypothesis suggests that the feeling of unease in the Uncanny Valley arises from our social and cultural context. If artificial human-like figures are perceived as strange or inappropriate in our society, they can evoke negative emotions. Study of Kao et al. (2019) finds that avatars with higher anthropomorphism led to higher player experience. Avatars with higher anthropomorphism also led players to identify more highly with their avatars. Independent of avatar type, we find avatar identification significantly promotes player experience. Players playing games doomed by little humanoidness. We will be more successful when the avatar is more a human."

Point 3: At p. 3/13 the concept of utility is still unclear. First: "In real systems, chatbots [...] are able to perform more accurate peripheral search and identification tasks while performing a central search". I think there is a mistake here as it seems that are chatbots perfoming peripheral search and identification tasks. I guess the authors wanted to say that in real systems, given that chatbots are usually located peripherally, they require the users to perform a peripheral search while performing a central task (which is in line with the attention-utility issue discussed by McCrickard and colleagues). The following phrase "Mimicked expressions of emotions are signals of high biological value" does not link with the one before and the one after and drift attention from the definition of the utility concept. I would suggest removing it. The authors then discuss utility, but its definition is still hard to catch. Utility refers to a system's usefulness to its users or customers. What McCrickard and colleagues discuss

in the context of peripheral system design, is that utility refers to the ways in which peripheral cues or messages help users accomplish their goals without requiring their full attention. Therefore, the sentence "Utility, like usability, can be evaluated as an aspect of human-computer interaction for the purpose of identifying aspects of this interaction that can be improved with the help of evaluation methods" still doesn't capture the definition of utility. Also, the difference in the conceptualization of utility between McCrickard and colleagues, and the one provided by the authors is unclear. What are the authors referring to when they talk about "individual gaps"? Once addressed this issue, I would place this section after the definition of utility for better clarity.

Response 3: We agree with the Reviewer remarks and we have implemented their suggestions. Indeed, there was a mistake with subjects in the sentence, which disrupted the logical flow. Thank you for identifying that. We have worked on the corresponding excerpts to make them more clear. We have referred to the general understanding of utility and referred to the works of McCrickard and colleagues, adding an extra citation (McCrickard and Chewar) as well elaborated on what we understand by individual gaps in the context of utility.

Point 4: At p. 3/13 there is a "s" after the [25].

Response 4: The redundant letter has been removed.

Point 5: At p. 9/13 there is a missing in-text reference number (McCrickard et al.).

Response 5: Missing quote has been corrected.
